# Clinical and ultrasonographic features of choroidal metastases based on primary cancer site: Long-term experience in a single center

**DOI:** 10.1371/journal.pone.0249210

**Published:** 2021-03-25

**Authors:** Maria Antonietta Blasi, Martina Maceroni, Carmela Grazia Caputo, Maria Grazia Sammarco, Andrea Scupola, Jacopo Lenkowicz, Giovanni Schinzari, Ernesto Rossi, Monica Maria Pagliara

**Affiliations:** 1 Università Cattolica del Sacro Cuore, Rome, Italy; 2 UOC Oncologia Oculare, Fondazione Policlinico Universitario A. Gemelli - IRCCS, Rome, Italy; 3 Fondazione Policlinico Universitario A. Gemelli IRCCS, Rome, Italy; 4 Oncologia Medica, Fondazione Policlinico Universitario, A. Gemelli IRCCS, Rome, Italy; Bascom Palmer Eye Institute, UNITED STATES

## Abstract

**Introduction and purpose:**

Choroidal metastases (CM) are the most common intraocular malignancies. With longer survival rates for cancer patients, CM will be increasingly encountered. We evaluated clinical and ultrasonographic (US) characteristics of CM in order to identify diagnostic biomarkers that correlate with the primary tumor site.

**Methods:**

The medical records of all patients with CM evaluated at the Ocular Oncology Unit between February 2010 and March 2020 were analyzed.

**Results:**

82 eyes of 70 patients were included. The primary cancer site was lung in 26 patients (37%), breast in 23 (33%), kidney in 9 (13%), gastrointestinal in 5 (7%), thyroid in 5 (7%), parathyroids and prostate respectively in 2 (3%). Fifty-five patients (78%) had other systemic metastases at the time of ocular diagnosis. Ten (14%) patients had no history of primary cancer. Bilateral CM were found in 20 patients (29%); fifty-six eyes (68%) had a single CM. The epicenter of CM was predominantly macula (43 eyes, 52%). The mean thickness was 4,1 mm (range 1,8–12,3). US structure was inhomogeneous in 67 eyes (82%). Reflectivity was mainly medium (39%) and medium-low (39%). In particular, CM from lung cancer showed lower reflectivity than those from the breast (p = 0,02). CM deriving from lung cancer were typically dome-shaped, whereas CM originating from breast were characteristically plateau shaped (p = 0,02). Seventy-four (91%) eyes presented fluid on optical coherence tomography.

**Conclusion:**

We significatively found that CM from lung cancer generally appear dome-shaped with medium-low internal reflectivity, whereas those from breast cancer typically present a plateau appearance and higher internal reflectivity. Though it is hard to identify the site of the primary tumor relying exclusively on clinical and US aspects, morphology and internal reflectivity can be considered as diagnostic biomarkers. Thus, the origin of the primary tumor can be suspected by integrating a constellation of findings.

## Introduction

With the population ageing, the overall incidence of cancer continues to rise and, as the treatment options are improving, the mortality rates from cancer are declining. Therefore, it is likely that patients with uveal metastases will be increasingly encountered. Uveal metastases are the most common intraocular malignancies and up to 10% of patients with metastatic cancer have intraocular involvement [1, 2]. The choroid is the most common site of the uveal tract involved by metastases because the hematogenous dissemination of tumor emboli from remote sites often leads to choroidal vasculature [3, 4]. Most choroidal metastases (CM) originate from breast cancer in women and lung cancer in men [3–5]. Generally, at the ophthalmoscopic examination CM appear as creamy white or yellow masses, dome or plateau shaped, usually associated with subretinal fluid. They can present as unifocal or multiple, with unilateral or bilateral involvement. The diagnostic tests include Ultrasonography (US), a useful tool required to evaluate tumor size, shape, and location and to estimate reflectivity, internal structure and degree of vascularization. Recently, enhanced depth imaging optical coherence tomography (EDI-OCT) and OCT angiography (OCT-A) have shown to play a role in the evaluation of metastases [6].

We retrospectively reviewed our data on CM, providing an assessment of clinical and ultrasonographic features. As color appearance has been suggested to be a way to identify the site of primary cancer [7–9], in this study we evaluated additional clinical signs and ultrasonographic characteristics of CM in order to find a correlation with the primary tumor. These data would be particularly useful in the differential diagnosis or in the absence of any detectable primary tumor.

## Patients and methods

The medical records of all patients with a clinical diagnosis of CM evaluated at the Ocular Oncology Unit of Fondazione Policlinico Gemelli between February 2010 and March 2020 were analyzed.

Patients’ medical records were accessed between January 2020 and April 2020. Eyes with missing or incomplete clinical or US records were excluded.

We collected information on the primary tumor site, primary cancer histological type, interval (months) between primary tumor diagnoses and ocular diagnoses, the presence of other systemic metastases. The patients’ age at diagnosis of ocular metastases and their gender were recorded. Information as the involved eye (right or left), laterality (unilateral or bilateral), and the number of metastatic foci (single metastases, multiple metastases) were registered. The anteroposterior location of the epicenter of the largest choroidal metastasis (macula, between the macula and the equator and anterior to the equator) was described. The presence of subretinal fluid detected with Optical Coherence Tomography (OCT) was reported.

The US features such as tumor thickness, internal reflectivity, acoustic structure, ultrasonographic shape, and the presence of associated retinal detachment were recorded. Internal tumor reflectivity was assessed qualitatively using B-scan and quantitatively using A-scan and graduated in five categories according to Type I quantitative ultrasonography classification by Ossoinig: low, medium-low, medium, medium-high, and high. The acoustic structure was analyzed in terms of homogeneous or inhomogeneous, relying on B-scan images and A-scan intralesional spikes. Tumor shape on US was registered as dome-shaped, mushroom-shaped, or plateau-shaped. Associated retinal detachment or shallow epilesional subretinal fluid detectable on US were reported.

The study was approved by the ethics committee of Fondazione Policlinico Universitario A. Gemelli. All data were fully anonymized before analysis. All patients gave informed written consent for medical records to be used in medical research.

### Statistics

All data were tabulated on Microsoft Excel 2018. The percentage distribution by gender, ethnicity, and qualitative parameters was calculated; measures of central tendencies (mean, range) were obtained for quantitative parameters. The correlation between primary tumor site and clinical and ultrasonographic features was analyzed using the chi-squared test for categorical variables and the t-test or Wilcoxon-Mann-Whitney test for continuous variables. Correlation analyses were conducted by filtering the cohort of patients for breast and lung, which were by far the most numerous categories for the variables under study.

## Results

Among the 87 eyes of 75 analyzed patients, 82 eyes of 70 patients met all the requirements of the inclusion/exclusion criteria. Patients’ demographic characteristics are listed in Table 1. Mean age and gender distribution based on primary cancer site are listed in Table 2.

**Table 1:** Patients’ demographics.

Patient, n	Age at ocular diagnosis, mean (range)	M/F, n (%)	Caucasian Race, n (%)
70	61 (30–85)	31 (44) / 39 (56)	70(100%)

**Table 2:** Patients’ characteristics based on the primary cancer site.

	Lung	Breast	Kidney	Gastro-intestinal	Thyroid	Parathyroids	Prostate	Total Or Mean
N. of Patients analyzed (%)	26 (37)	23 (33)	9 (13)	5 (7)	5 (7)	1 (1.5)	1 (1.5)	70 (100)
N. of Eyes (%)	31 (38)	29 (35)	10 (12)	5 (6)	5 (6)	1 (1.5)	1 (1.5)	82 (100)
Age (range)	61 (39–85)	56 (30–75)	67 (49–82)	60 (47–80)	64(32–79)	60	80	61 (30–85)
Gender (M/F)	14/12	0/23	9/0	4/1	2/3	1/0	1/0	31/39

F = female, M = male.

There were 31 males (44%) and 39 females (56%), with a mean age of 61 (range 30–85) at the time of ocular diagnoses. The primary cancer site was lung in 26 patients (35%), breast in 23 patients (32%), kidney in 9 patients (12%), gastrointestinal in 5 patients (6%), thyroid in 5 patients (6%), parathyroids and prostate respectively in the remaining 2 patients (3%).

Fifty-five patients (78%) had other systemic metastases when the ocular diagnosis was done. Systemic metastases were present in 95% of patients with breast carcinoma and in 49% of patients with lung carcinoma (p < 0.01) at the time of ocular diagnosis. Ten (14%) out of 70 patients had no history of primary cancer; all of them received a diagnosis of lung primary tumor after a complete oncologic examination. In the remaining 60 patients (86%), ocular metastases were detected after the diagnosis of the primary tumor. In particular, CM were found after a mean of 57 months (range 0–280) from primary cancer diagnose. Aside from the patients for whom the ocular diagnosis was the first manifestation of unknown cancer, all the other patients had received systemic chemotherapy or were receiving systemic chemotherapy at the time of ocular diagnosis.

Bilateral choroidal involvement was found in 20 patients (29%); unilateral choroidal involvement was found in 50 patients (71%). Fifty-six eyes (68%) had a single metastasis, while 26 eyes (32%) had multiple metastatic foci (from 2 to 7 foci). The anteroposterior location of the epicenter of the tumor was the macula in 43 eyes (52%), between the macula and the equator in 32 eyes (39%), and anterior to the equator in 7 patients (9%). Clinical characteristics of uveal metastases in relation to the primary cancer site are summarized in Table 3.

**Table 3:** Primary tumor and eye metastases characteristics based on primary cancer site.

82 eyes included	Lung	Breast	Kidney	Gastro-intestinal	Thyroid	Parathyroids	Prostate	Total
Carcinoma/carcinoid	24/7	29/0	10/0	5/0	5/0	1/0	1/0	75/7
Other metastases Yes/no	19/12	28/1	9/1	4/1	3/2	1/0	0/1	64/18
Eye Met. Single/multiple	21/10	16/13	8/2	5/0	4/1	1/0	1/0	56/26
Bilateral/Unilateral	11/20	15/14	2/8	0/5	3/2	0/1	0/1	31/51
Ora serrata-equator/equator-macula/macula	1/11/19	3/10/16	3/5/2	0/2/3	0/4/1	0/0/1	0/0/1	7/32/43
Mean interval (range) from primary tumor diagnose and CM	22 (0–84)	92 (12–280)	94 (12–252)	87 (36–240)	24 (12–36)	72	12	0–280

The mean visual acuity in the affected eye was 20/50 Snellen Equivalent (range 20/800-20/20).

**Fig 1:**
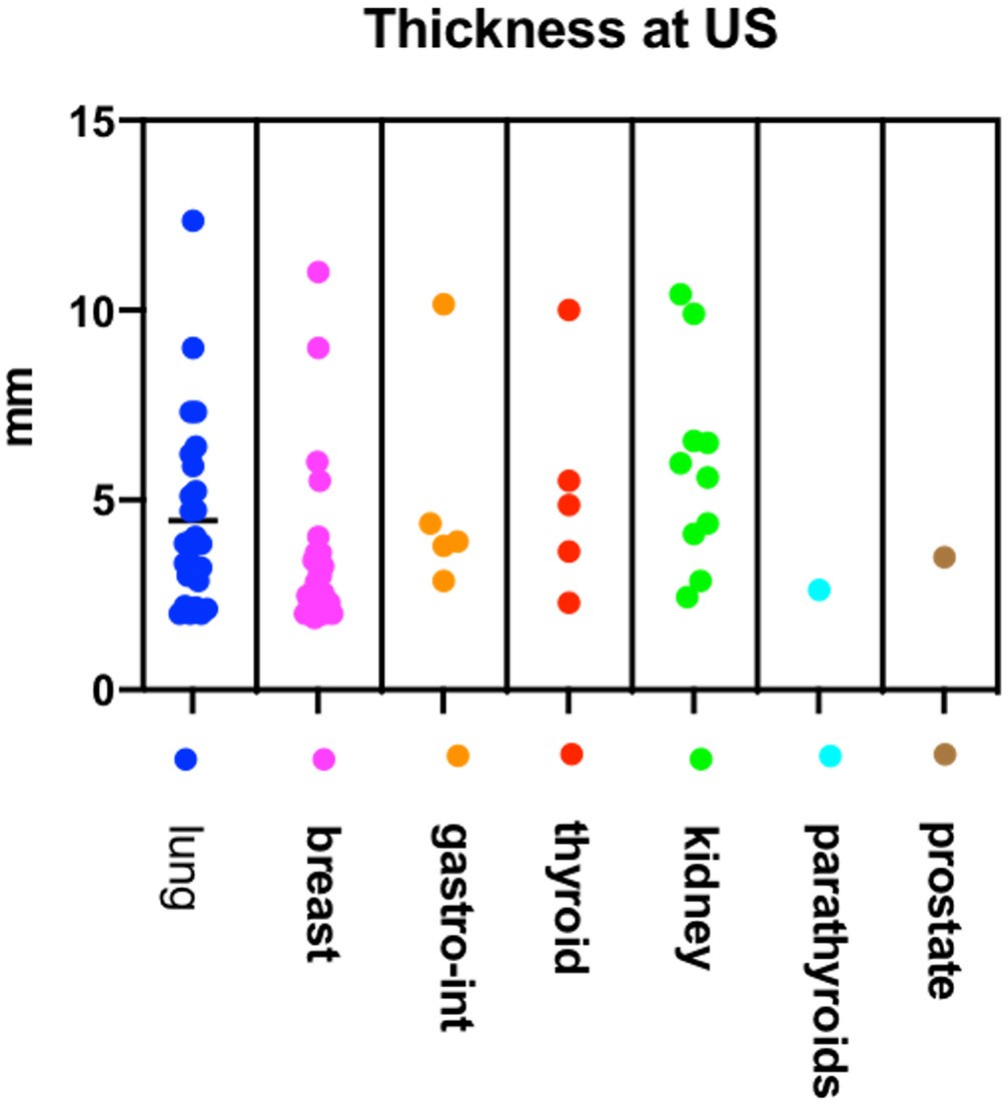
US thickness of CM detected on US based on primary cancer site. The nested graph illustrates the thickness of CM detected on US based on the primary cancer site. No statistically significant correlation was found between CM thickness and the primary tumor site.

The mean thickness value calculated with ultrasonography was 4,3 mm (range 1,8–12,3) in eyes with metastases from lung cancer, 3,3 mm (range 1,9–11) in breast cancer, 6,5 mm in kidney cancer (range 2,8–10,4), 5,02 mm (range 2,8–10,1) in gastrointestinal cancer, 5,2 mm (range 2,3–10) in thyroid cancer and 2,6 and 3,4 respectively in metastases from parathyroids and prostate cancer (Fig 1).

**Fig 2:**
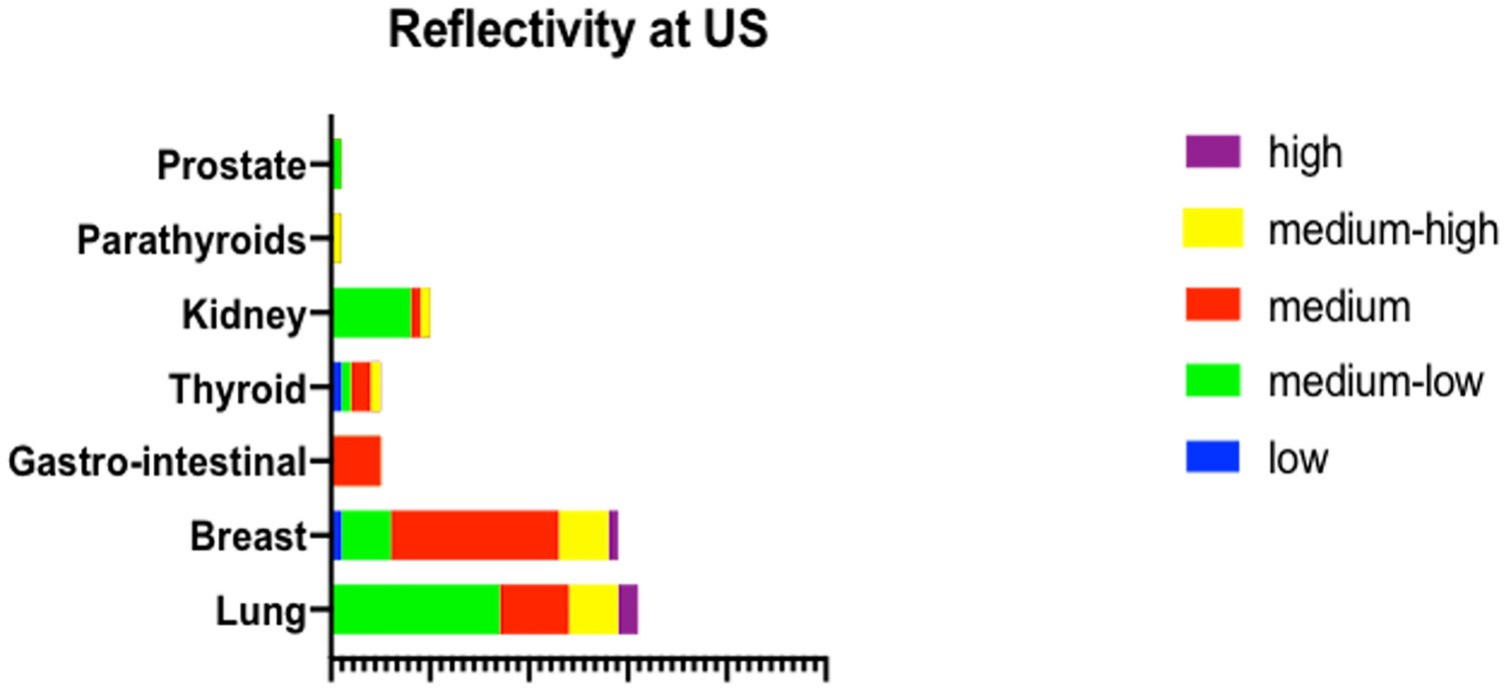
US reflectivity of CM detected on US based on primary cancer site. The grouped bar graph illustrates the reflectivity of CM detected on US based on the primary cancer site. CM arisen from lung cancer showed a lower reflectivity if compared to CM developed from breast cancer (p = 0,02).

**Fig 3:**
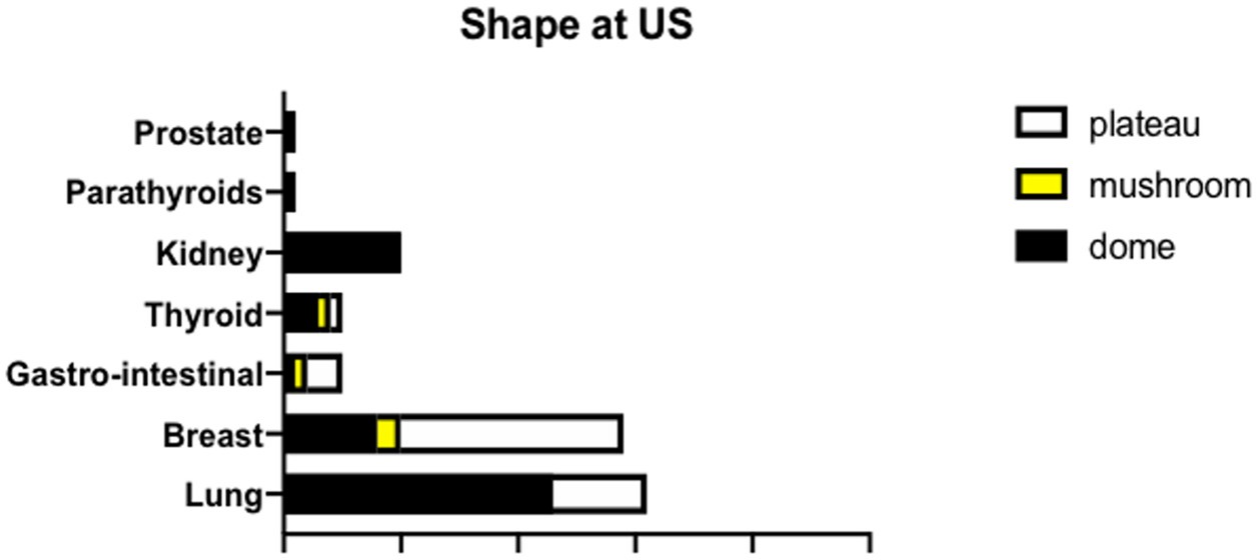
US morphology of CM based on primary cancer site. The grouped bar graph illustrates the morphology of CM detected on US based on the primary cancer site. CM from lung carcinoma were typically dome-shaped, while CM from breast carcinoma were characteristically plateau-shaped (p = 0,02).

The ultrasonographic structure was registered as homogeneous in 15 eyes (18%) and inhomogeneous in 67 eyes (82%). Reflectivity at ultrasonography (Fig 2) was high in 3 eyes (4%), medium-high in 13 eyes (16%), medium in 32 eyes (39%), medium-low in 32 eyes (39%) and low in 2 eyes (2%). In particular, CM arisen from lung cancer showed a lower reflectivity if compared to CM developed from breast cancer (p = 0,02). Forty-seven eyes (57%) showed a dome-shaped morphology on US, whereas 31 (38%) and 4 eyes (5%) presented respectively a plateau-shaped and a mushroom-shaped appearance (Fig 3). CM deriving from lung cancer were typically dome-shaped, whereas CM originating from breast cancer were characteristically plateau-shaped (p = 0,02). Sixty eyes (73%) showed epilesional or positional retinal detachment on US, whereas 74 eyes (91%) presented subretinal fluid at optical coherence tomography (OCT). The ultrasonographic features of CM in relation to the primary cancer site are reported in Table 4.

**Table 4:** The US features based on primary cancer site.

	Lung	Breast	Kidney	Gastro-intestinal	Thyroid	Parathyroids	Prostate	Mean or Total
**Thickness** mm (range)	4.3(1.8–12.3)	3.3(1.9–11)	6.5(2.8–10.4)	5,02 (2.8–10.1)	5.2(2.3–10)	2.6	3.4	4.2 (1.8–12.3)
**Structure:** homogeneous/inhomogeneous	5/26	4/25	3/7	1/4	2/3	0/1	0/1	15/67
**Reflectivity**								
*Low*	0	1	0	0	1	0	0	2
*Medium-low*	17	5	8	0	1	0	1	32
*Medium*	7	17	1	5	2	0	0	32
*Medium-high*	5	5	1	0	1	1	0	13
*High*	2	1	0	0	0	0	0	3
**Morphology**								
*D*ome	23	8	10	1	3	1	1	47
*Mushroom*	0	2	0	1	1	0	0	4
*Plateau*	8	19	0	3	1	0	0	31

## Discussion

CM represent the most common uveal malignancy, being more common than primary uveal melanoma. We reported our data on CM, providing an evaluation of clinical and ultrasonographic characteristics in relation to the primary tumor site.

### Primary tumor site

According to the published literature, the primary cancers that most commonly lead to uveal metastases include breast cancer (40–47%) and lung cancer (21–29%) [3–5]. In our series, the most common primary tumor sites were lung and secondly breast, with a small difference in incidence (35% vs 32%). As far as gender is concerned, we found that CM occurred more commonly in females -*n (%)* = 39 (55%) -, which is likely attributable to the frequent occurrence of breast metastasis to the uvea, embodying 23 patients overall with the total of cases in females. The incidence of breast cancer is increasing steadily, and currently, one in every eight women is expected to develop this malignancy during her lifetime [10].

Forty-six % of patients affected by lung cancer were women. While lung cancer has historically affected primarily men, the gap between genders is narrowing quickly. The rise and growing epidemic status of lung cancer in women is overwhelmingly attributed to tobacco use, albeit the rank of the disease in nonsmokers suggests that other factors such as geographic-cultural, genetic, hormonal, and possibly infectious factors can play an etiologic role [11, 12].

In our series, all those primary cancers discovered after the CM were lung cancers, underlining the propensity of lung carcinoma to metastasize early and suggesting that presence of CM in patients without a history of cancer should prompt a thorough investigation for lungs, especially in males.

In addition, our study revealed that the mean interval between primary cancer diagnosis and CM is consistently shorter for lung than for breast -n of months (mean) = 22 vs 92 respectively-, confirming literature evidence [5, 13–16].

At the time of ocular diagnosis, systemic metastases were present in 95% of patients with breast carcinoma and only in 49% of patients with lung carcinoma (p < 0.01) (Table 5), corroborating that choroidal involvement typically occurs late in the course of breast cancer disease [5, 17].

**Table 5:** Statistically significant correlations between clinical and US parameters and primary tumor site.

	Breast vs Lung	P-value
Presence of other systemic metastases at the time of ocular diagnoses	95% vs 49%	**< 0.01**
Shape on US	Plateau vs Dome	**0,02**
Reflectivity on US	Medium-high vs Medium-low	**0,02**
Histology	Carcinoma vs Carcinoid	**0,02**
Number of CM	Single vs Multiple CM	0,4
Ocular involvement	Unilateral vs bilateral	0,3
Acoustic structure	Inhomogeneous vs Homogeneous	1
Presence of Fluid	Yes vs no	0.6

Regarding CM from kidney, GI, thyroid, parathyroids, and prostate, all the ocular metastases were diagnosed after primary tumor diagnosis, thus representing the manifestation of systemic widespread cancer. The mean interval between primary cancer diagnosis and CM ranged from 12 to 252 months, with the kidney having the mean longest interval [8, 18–20].

It is interesting to note that CM from GI and prostate were found in 6% and 1.5% of our patients respectively. In Europe, prostatic cancer represents 21% of all cancers and 10% of all cancer deaths in men, whereas colorectal cancer represents 12.8% of all cancers and 12.6% of cancer-related deaths in both sexes [21]. This discrepancy in incidence between the primary tumor and the CM could be due to biological factors related to the primary site and responsible for flourishing of metastatic cells in tissue in which they arrive.

### Clinical features

Data from our series revealed that CM from lung cancer appeared typically single and unilateral, whereas CM from breast cancer were single and in 51% of cases bilateral, albeit these results were not statistically significant. In a review of 520 eyes, Shields et al. [4] reported that metastases from the lung were more often unilateral and unifocal. Although previous reports had described CM from breast cancer as bilateral and multifocal, Demirci et al. [5] and Kostantinidis et al. [14] found unilateral ocular involvement respectively in 63% and 81% of patients with breast cancer.

Coherently with the literature findings, CM from kidney and gastro-intestinal cancer presented as single and unilateral (in 90% and 100% of patients respectively), whereas thyroid cancer was predominantly associated with bilateral manifestation. Metastasis from prostate cancer appeared as single and unilateral, confirming the available evidence [14, 22], as well as metastases from parathyroids. Unfortunately, due to the paucity of specific data regarding parathyroids, it is difficult to derive conclusions about their typical clinical presentation.

Similar to other studies [23], we found that more than 90% of intraocular metastases were post-equatorial. Ferry and Font [3] speculated that the posterior uveal distribution of metastases was related to the abundant supply of posterior ciliary arteries to the choroid. Regarding tumor appearance at fundoscopy, 10 out of 82 eyes showed orange-colored CM. Sixty % derived from lung carcinoid, whereas 40% originated from kidney cancer, confirming what was postulated by Harbour et al., [7] and Haimovici [8]. Aside from these exceptions, all other metastases were yellowish in color.

### US features

Few published works have specifically explored the topic of Ultrasonography (US) in CM, mainly focusing on metastases in general and their differences with choroidal melanoma, without deepening the specific US appearance in relation to the primary cancer site. Identifying US biomarkers correlating with the primary cancer site appears to be of particular interest, especially because ultrasound is a non-invasive technique.

On B-scan US, CM appear mainly plateau-shaped, less frequently dome-shaped, and rarely mushroom-shaped; on A-scan US, CM generally reveal high or medium reflectivity, with an internal V-shaped pattern for thicker metastases [24, 25]. Internal vascularization is usually minimal or absent and serous retinal detachment is often detected.

**Fig 4:**
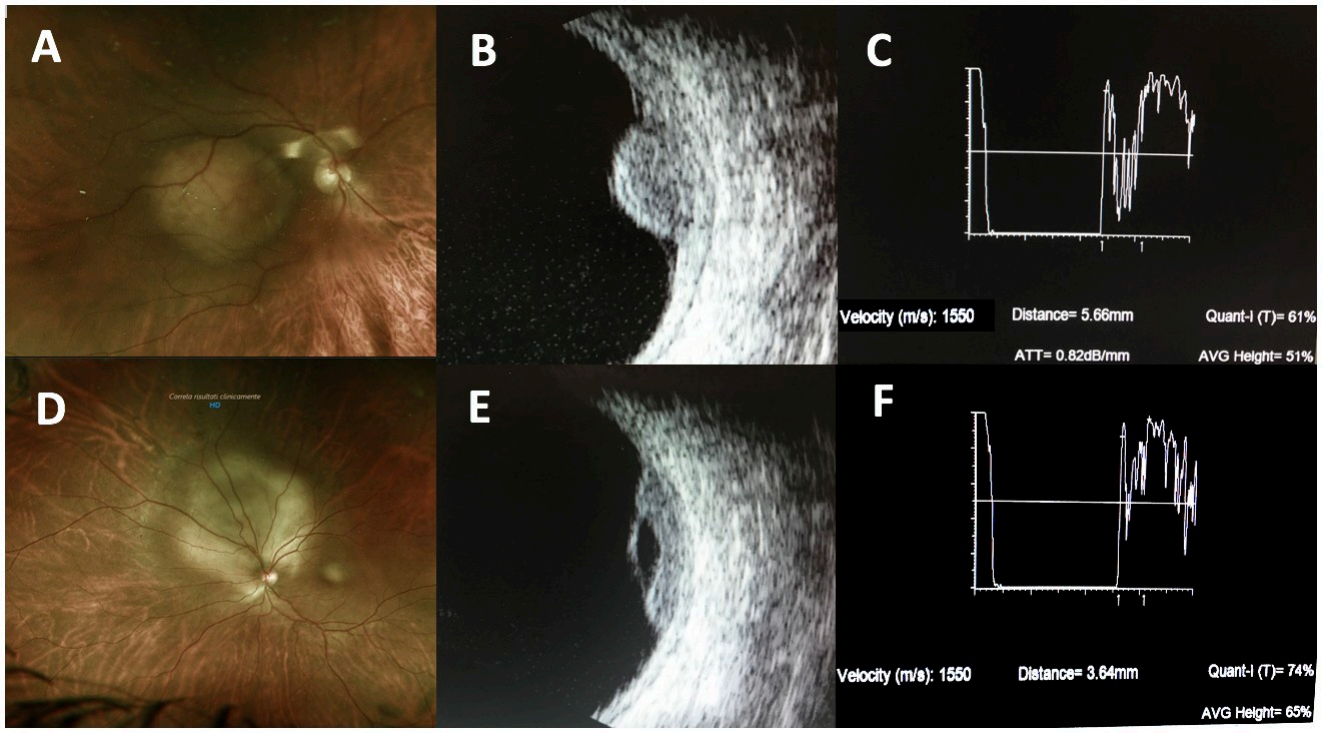
Typical ophthalmoscopic and US appearance of CM from lung cancer (respectively A and B-C) and breast cancer (respectively D and E-F). CM originating from lung cancer generally appear as dome-shaped lesions with medium-low internal reflectivity, whereas those from breast cancer typically present a plateau and irregular appearance and higher internal reflectivity.

Analyzing our data, CM from lung carcinoma appeared typically dome-shaped, while CM from breast carcinoma were characteristically plateau-shaped (p = 0,02) (Fig 4A, 4B, 4D and 4E, Table 5). Shields et al. reported that CM from the breast are plateau-shaped in 80% of cases, whereas CM from lung cancer are plateau-shaped in 56% of cases and dome-shaped in the remaining 44% [4].

In our series, a mushroom appearance was found in 4 large metastases with a diffuse pattern, 1 measuring 5.5 mm and 3 more than 9 mm in maximum thickness. Tumor primary site was breast in 2 cases, GI and thyroid in 1, although in literature mushroom appearance due to rupture of the Bruch membrane has been predominantly associated with lung cancer [14]. The “mushroom” configuration probably reflects the rapid vertical growth and the large size of the tumors [25].

Furthermore, two peculiar cases originating from breast cancer showed a diffuse choroidal infiltration associated with a total retinal detachment: a diffuse pattern is typically associated with breast cancer, representing the clinical presentation of about 4% of cases [5].

Confirming what was reported by Shields et al. [4], flatter tumors were observed with CM from breast cancer (3.3 mm), while thicker tumors were observed with metastases originating from the kidney (6.5 mm) and lung cancers (4.3 mm).

As reported by Sobotka et al, in general CM are characterized by a medium-high reflectivity [26]. By A-B scan ultrasonography, CM arisen from lung cancer showed a lower reflectivity if compared to CM developed from breast cancer (p = 0,02) (Fig 4B, 4C, 4E and 4F, Table 5). The acoustic profile of CM is explained by the histoarchitecture of the tumor, as CM from the breast has solid epithelial nests or glandular structures, which act as echo-producing interfaces, resulting in high reflectivity and an irregular internal structure on US [27]. The different intralesional reflectivity probably reflects the different internal cytoarchitecture.

It is worth mentioning that 3 out of 10 ocular masses from kidney cancer presented anecogenous cysts. Although cavitation is suggestive of uveal melanoma, it has been reported in several CM of renal origin. In fact, cystic cavitation can be due to intralesional necrosis, hemorrhage or accumulation of mucus-proteinaceous substances [28].

It is interesting to investigate the ability of US to detect fluid in comparison to OCT. Sixty eyes (73%) showed epilesional or positional retinal detachment on US, whereas 74 eyes (91%) presented subretinal fluid at OCT, confirming a greater sensitivity of OCT in fluid detection.

## Conclusion

We reported the ten-years experience of the Ocular Oncology Unit on CM to identify a correlation between clinical and ultrasonographic features and the primary cancer site.

The main limit of the study is the small sample size. However, we believe that the collection of patients with CM from different primary cancer sites provides a comprehensive overview of the topic and represents a strength of the study.

Though it is hard to identify the site of the primary tumor relying exclusively on clinical and US aspects, in some instances, the origin of the primary tumor can be suspected by integrating a constellation of findings.

Although bilateral involvement is strongly suggestive for secondary lesions, CM are frequently unilateral and unifocal. Even though CM are paradigmatically considered to have medium-high reflectivity on US, they often present with a medium or medium-low reflectivity, thus masquerading choroidal melanoma and causing significant diagnostic confusion.

We found a statistically significant association between US morphology and reflectivity and the primary cancer site for lung and breast cancer. CM from lung cancer generally appear dome-shaped with medium-low internal reflectivity, whereas those from the breast are typically plateau-shaped with higher internal reflectivity. Therefore, morphology and reflectivity can be considered as diagnostic biomarkers to orient in the detection of the primary tumor.

Multicentric studies are needed to elucidate the relation between CM features and rarer primary tumor sites.

## Supporting information

S1 Data (XLSX)
